# Synthetic Activation of Endogenous PI3K and Rac Identifies an *AND*-Gate Switch for Cell Polarization and Migration

**DOI:** 10.1371/journal.pone.0003068

**Published:** 2008-08-27

**Authors:** Takanari Inoue, Tobias Meyer

**Affiliations:** Chemical and Systems Biology, Bio-X Program, Stanford University, Stanford, California, United States of America; The Beatson Institute, United Kingdom

## Abstract

Phosphatidylinositol 3-OH kinase (PI3K) has been widely studied as a principal regulator of cell polarization, migration, and chemotaxis [1], [2], [3], [4]. Surprisingly, recent studies showed that mammalian neutrophils and *Dictyostelium discoideum* cells can polarize and migrate in the absence of PI3K activity [5], [6], [7]. Here we directly probe the roles of PI3K and its downstream effector, Rac, in HL-60 neutrophils by using a chemical biology approach whereby the endogenously present enzymes are synthetically activated in less than one minute [8], [9], [10]. We show that uniform activation of endogenous PI3K is sufficient to polarize previously unpolarized neutrophils and trigger effective cell migration. After a delay following symmetrical phosphatidylinositol (3,4,5)-triphosphate (PIP_3_) production, a polarized distribution of PIP_3_ was induced by positive feedback requiring actin polymerization. Pharmacological studies argue that this process does not require receptor-coupled trimeric G proteins. Contrary to the current working model, rapid activation of endogenous Rac proteins triggered effective actin polymerization but failed to feed back to PI3K to generate PIP_3_ or induce cell polarization. Thus, the increase in PIP_3_ concentration at the leading edge is generated by positive feedback with an *AND* gate logic with a PI3K-Rac-actin polymerization pathway as a first input and a PI3K initiated non-Rac pathway as a second input. This *AND*-gate control for cell polarization can explain how Rac can be employed for both PI3K-dependent and -independent signaling pathways coexisting in the same cell.

## Introduction

Chemotactic cells such as neutrophils have the ability to sense, orient themselves and migrate toward a chemoattractant source even if the chemoattractant concentration difference is as small as a few per cent across the cell [11]. Understanding the molecular mechanism underlying this remarkable sensing capability is a fundamental question of chemotaxis research. In 1972, Alfred Gierer and Hans Meinhardt proposed an elegant conceptual model explaining how spatial patterns can be formed during development [12]. They later adapted the same concept to propose a framework for directional sensing of neutrophils and other chemotactic cells [13]. In their model, a self-enhancing positive feedback type reaction is triggered locally inside the cell where the chemoattractant concentration is highest and thereby orients the cell towards the source of the chemoattractant. The triggering of this local positive feedback is followed by a negative feedback that either globally suppresses the self-enhancing reaction or depletes activators from the back. The negative feedback is necessary to prevent the self-enhancing reaction to take place in other regions of the cell. The elegant logics of this model is that cells first polarize, reorganize the actin based migration machinery to the front and chemotax towards the source of the chemoattractant all based on the same local positive feedback loop [13]. This raised the question what molecular entities may constitute such a local positive feedback.

**Figure 1:**
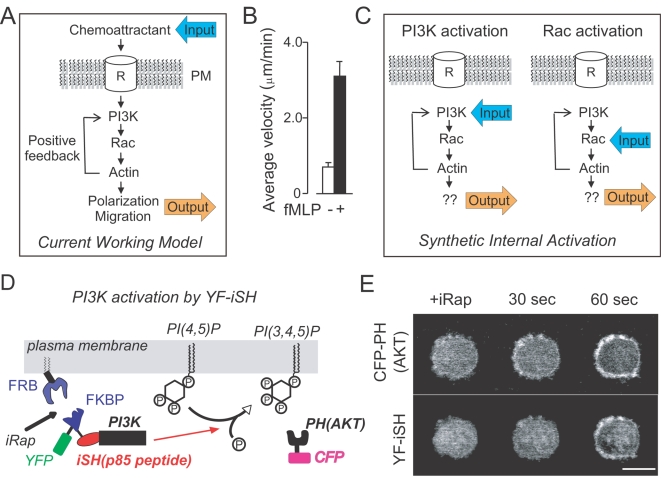
Synthetic activation of endogenous PI3K in HL60 neutrophil cells. (A) Working model for a local positive feedback loop that triggers cell polarization, migration and chemotaxis. (B) fMLP-induced cell migration was quantified by analyzing cell velocity before and after fMLP addition to differentiated HL-60 cells. Mean±S.E.M. (p<0.0001, n = 16 from two independent experiments). (C) Schematic representation of the synthetic activation of endogenous PI3K and Rac. If the feedback model is correct, activation of either endogenous PI3K or Rac should trigger polarization and migration. (D) Illustration of the synthetic PI3K activation system. iRap brings p85 derived YF-iSH together with the catalytic subunit of PI3K, p110, to the plasma membrane. Due to the induced proximity of PI3K to PIP_2_ substrates in the plasma membrane, PI3K produces PIP_3_ by phosphorylating PIP_2_. (E) Dual color fluorescent images of differentiated HL-60 cells show that iRap-induced YF-iSH translocation leads to rapid production of PIP3. Cells are shown every 30 seconds before and after iRap addition. Top: CFP-PH(AKT), Bottom: YF-iSH. Scale bar, 10 µm.

PI3K has been widely studied as a principal regulator of cell polarization, migration and chemotaxis [1], [2], [3], [4]. In addition, the discovery that PIP_3_ lipid second messengers are polarized from the front to back in migrating Dictyostelium cells [14], [15], neutrophils [2] and fibroblasts [16] suggested that PIP_3_ might be part of such a positive feedback loop. This was supported by experimental evidence that extracellular addition of PIP_3_ analogs were shown to be sufficient to cause polarization and subsequent migration in neutrophils and neutrophil-like cells such as HL-60 cells [17], [18], [19]. There was also evidence that the introduction of exogenous PIP_3_ stimulates endogenous PIP_3_ production [19]. Together, these studies argued that PIP_3_ is part of a positive feedback loop that can polarize cells and cause them to migrate. Experimental evidence further implied that also Rac proteins [20] and polymerized actin [21], [22] are part of this positive feedback loop. For example, cells with overexpressed constitutively active Rac had elevated levels of PIP_3_ production, and treatment with Latrunculin A (LatA), an inhibitor for actin polymerization, led to an inhibition of the Rac-induced PIP_3_ production. This led to the plausible model that PI3K, Rac and polymerized actin are the three core components of a local positive feedback, explaining how cells can polarize, how they organize the cell migration machinery to the front and how they are directed towards the chemoattractant source (Figure 1A) [19], [20], [21]. Nevertheless, a number of recent studies have complicated this model since neutrophils and Dictyostelium cells were found to polarize, migrate, and chemotax in the absence of PI3K activity [5], [6], [7]. This raised important new questions such as whether this PI3K positive feedback really exists, whether endogenous PI3K is on its own sufficient to trigger cell polarization and migration and whether Rac, downstream of PI3K, may serve as an integrating mediator that can trigger polarized actin organization and cell migration on its own.

While positive feedback is believed to be a fundamental control principle that defines many, if not most, spatial and temporal signaling responses [13], [23], [24], the wiring of a particular positive feedback loop is inherently difficult to investigate since experimental perturbations have to be faster than the time of the feedback loop to prevent the feedback from enhancing or suppressing the perturbation. This argues that the most direct method to distinguish whether a signaling component is merely required for a signaling pathway or whether it functions as part of a positive feedback loop is to perform rapid chemical perturbations that are faster than the feedback time constants. Here we combined a rapid chemically induced perturbation strategy [8], [9], [10] with single-cell biosensors for different signaling steps to dissect the PI3K positive feedback module for neutrophil polarization and migration.

## Results and Discussion

**Figure 2:**
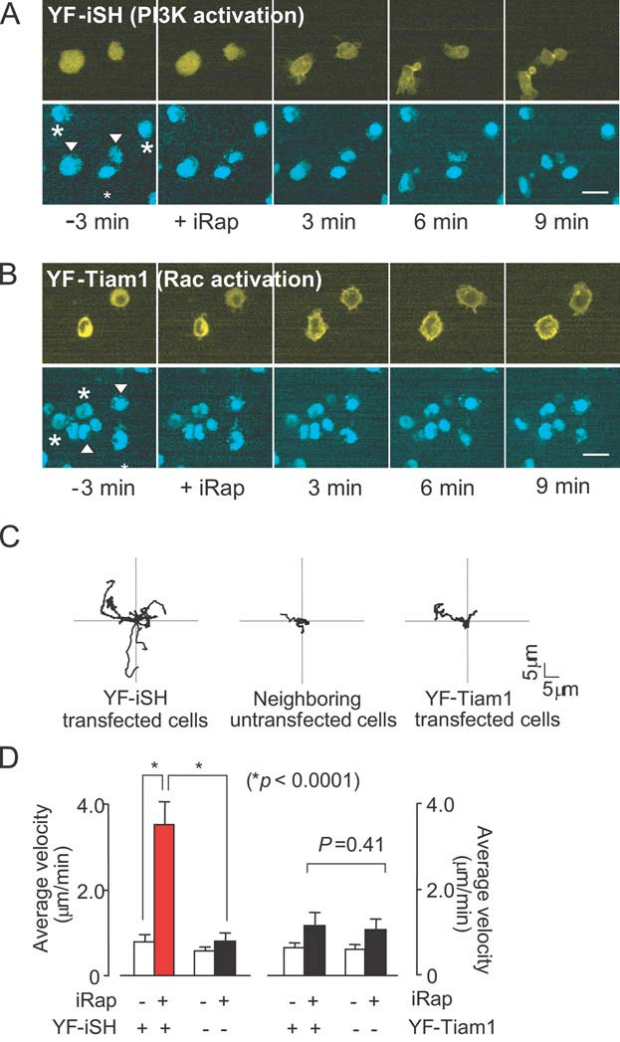
Synthetic activation of endogenous PI3K, but not of endogenous Rac, induces neutrophil polarization and migration. Dual color fluorescent images of differentiated HL-60 cells are shown every 3min before and after iRap addition. (A) Neutrophils polarize and begin to migrate in response to synthetic activation of PI3K. (B) Neutrophils induce non-polar pseudopods in response to synthetic activation of endogenous Rac and do not migrate. Synthetic activation of Rac was based on the forced translocation of catalytic Rac GEF activity (from the exchange factor TIAM). FKBP-YFP-TIAM construct (YF-Tiam1) was expressed instead of the YF-iSH construct. In both image series, the top rows shows the YFP-tagged activation peptides (YF-iSH for PI3K or YF-Tiam1 for Rac), and the bottom images show DyeCycle-labeled nuclei. Arrowheads in images of the nuclei point to the transfected cells, whereas asterisks mark untransfected cells. (C) Migration paths of 10 representative cells for each condition. The initial location was normalized to the origin. Cells after synthetic activation PI3K (left), untransfected control cells in the same field of view (middle), and cells after synthetic activation of Rac (right). (D) Average velocity of cells before and after PI3K activation or Rac activation. Graph shows the velocity at 1.5 min before and 6 min after iRap addition. Error bars, S.E.M. (n = 20 from four independent experiments). Scale bars, 10 µm.

We first tested whether unpolarized HL-60 neutrophils can break their morphological symmetry and start to migrate when they are stimulated by a uniform concentration of fMLP [25], a bacteria-derived chemoattractant that activates PI3K. Consistent with the presence of a self-polarizing positive feedback downstream of the fMLP receptor (Fig. 1A), HL-60-cells polarized and started to randomly migrate (Fig. 1B). We then tested whether uniform activation of PI3K, without any receptor input, causes cell polarization (Fig. 1C, left). To exclude potentially supramaximal stimulation from expression of constitutively active PI3K enzyme or external delivery of phosphoinositide lipids [17], [18], [19], we used instead a method to chemically activate endogenous PI3K activity. In this method, a chemical dimerizer is used to translocate a peptide that activates PI3K from the cytosol to the plasma membrane. The PI3K activator peptide was derived from the inter SH2 domain region of the protein p85 (Fig. 1D). This synthetic system consists of two constructs, a PI3K activation probe (YFP-FKBP-interSH2 or YF-iSH) that interacts with endogenous PI3K, and a membrane-targeted FRB (Lyn-FRB or LDR). Addition of the dimerizer, iRap, to HL-60 cells triggered a rapid plasma membrane translocation of YF-iSH, which was followed by a production of PIP_3_ within approximately 30 seconds (Fig. 1E). The PIP_3_ production was monitored in real time by the translocation of a fluorescent PIP_3_ biosensor, CFP-labeled PH domain from AKT (CFP-PH(AKT)). Strikingly, within a minute following synthetic PI3K activation, the cells polarized and started to migrate randomly (Fig. 2A, Movie S1). This demonstrated that uniform activation of endogenous PI3K is sufficient to trigger symmetry breaking as well as cell migration.

If PI3K is sufficient for triggering polarization and if Rac and PI3K proteins are both part of the same positive feedback loop, then uniform activation of native Rac should be equally effective in triggering polarization and migration (see scheme in Fig. 1C, right). While previous experiments tested for a role for Rac by microinjection or by overexpression of a constitutively active form of Rac, we activated instead endogenous Rac by using the chemically-induced translocation of a Rac specific guanine nucleotide exchange factor, Tiam1 [8]. Markedly, synthetic activation of endogenous Rac led to rapid and symmetric pseudopod formation and ruffling along the cell periphery, but did not result in cell polarization or significant migration (Fig. 2B, Movie S2). The individual cell migration traces in Fig. 2C are from neutrophils whose starting coordinates were set to the same origin at t = 0 and were then tracked over time after synthetic activation of either endogenous PI3K (left) or endogenous Rac (right). Control experiments are shown for tracks of untransfected cells that did not undergo significant polarization and migration following iRap addition (middle).

**Figure 3:**
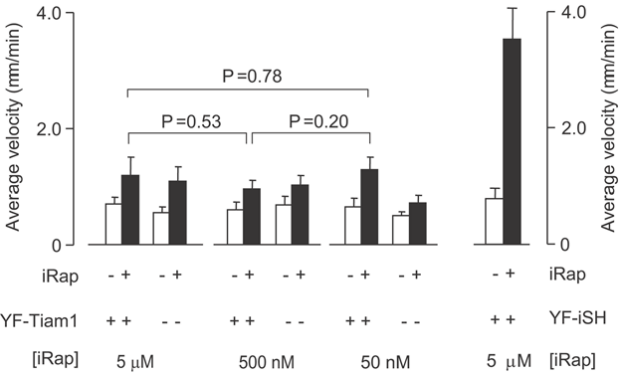
Control experiments show that neither maximal nor submaximal activation of endogenous Rac triggers cell polarization. Titration of the iRap concentration to determine whether submaximal Rac activation may trigger polarization and migration. Average velocities of Rac-activated cells was measured at different iRap doses (5 µM, 500 nM and 50 nM) before and after iRap addition. The right two bars show the average velocity of the PI3K-activated cells as a reference. Error bars are given as S.E.M. (n>15 from more than three independent experiments).

A quantitative analysis in Fig. 2D showed that induced cell migration by synthetic activation of PI3K is statistically highly significant (p<0.0001), while endogenous activation of Rac does not cause a significant increase in cell migration. We have previously found that receptor stimulation enhances subsequent Rac-induced PIP_3_ production in PC12 cells [26] and have tested if the same sensitization may occur in HL-60 cells. Differentiated HL-60 cells were first stimulated with a low dose of fMLP before we triggered iRap induced Rac activation. No significant Rac generated PIP_3_ was observed (data not shown), suggesting that HL-60 cells regulate their polarity machinery with a different mechanism. Since it has been reported that migration in fibroblasts works best for an intermediate range of Rac activation [27], we titrated iRap concentrations down from 5 µM to 50 nM. At no intermediate concentration did we observe polarization or migration following uniform Rac activation (Fig. 3). Thus, uniform activation of endogenous PI3K is sufficient to cause symmetry breaking and trigger migration while the symmetry breaking is lost at the level of Rac.

**Figure 4:**
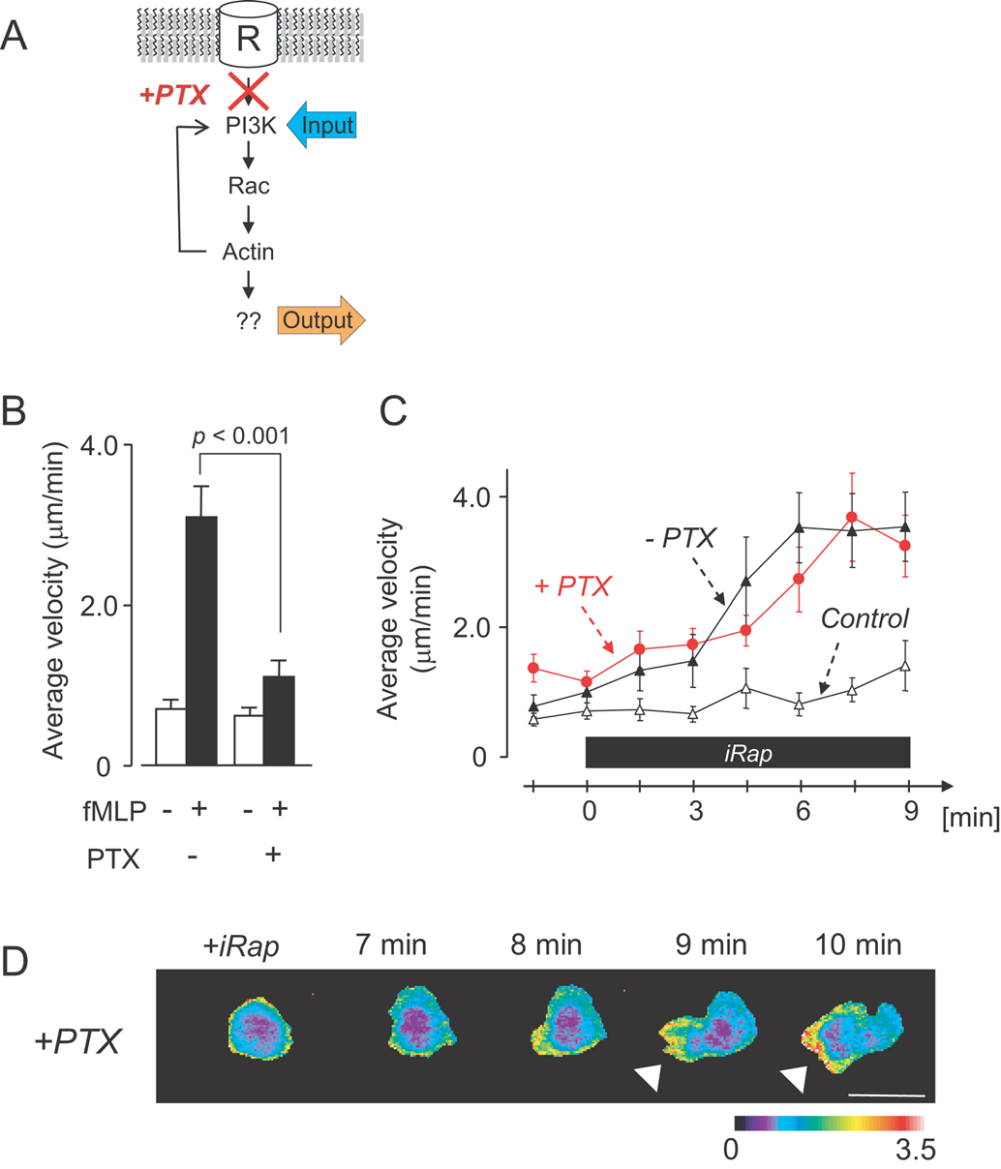
G-protein signaling is not required for PI3K-triggered cell polarization. (A) Schematic representation of experiments conducted in (B)–(D). (B) Control experiment showing that PTX suppresses fMLP-triggered cell migration. Differentiated HL-60 were stimulated with fMLP in the absence or presence of PTX. Average velocities are shown before and after fMLP addition (mean±S.E.M.; p<0.001, n = 16 from two independent experiments). (C) Comparison of the kinetics of the velocity increase following synthetic PI3K-activation in the presence or absence of PTX. Cells were transfected with the synthetic PI3K activation system and endogenous PI3K activated by iRap addition. Time axis is after iRap addition. PI3K-induced cell migration velocities increases are statistically indistinguishable in the presence (red circles) or absence (black triangles) of PTX, arguing that G-proteins do not contribute to the PI3K triggered polarity response. (Statistical analysis shows confidence values of 0.16<p<0.99; n≥16 from more than four independent experiments each). As a control, untransfected in the same field of view were analyzed. Error bars indicate S.E.M. (D) Time series of YF-iSH/CFP-PH(AKT) ratio image shows PIP_3_ accumulation (arrowheads) at the pseudopod of a PTX treated polarized cell. Scale bar, 10 µm.

To examine whether components upstream of PI3K might be part of the positive feedback loop triggered by PI3K activation, the synthetic activation of PI3K was repeated in the presence of Pertussis toxin (PTX), an inhibitor for the G protein that links chemoattractant receptor stimulation to PI3K activation [2] (Fig. 4A). In a control experiment, we first confirmed that PTX can suppress fMLP-induced migration (Fig. 4B). We then synthetically activated PI3K in PTX-treated cells and found that these cells can still polarize and migrate following PI3K activation. The velocities of PTX treated cells were also indistinguishable from control cells and increased over time with the same kinetics (Fig. 4C). Polarized cells also appeared to be morphologically indistinguishable (Fig. 4D). This data suggests that cell polarization and migration can be induced in cells through the activation of PI3K, bypassing the need for all upstream components in this system.

**Figure 5:**
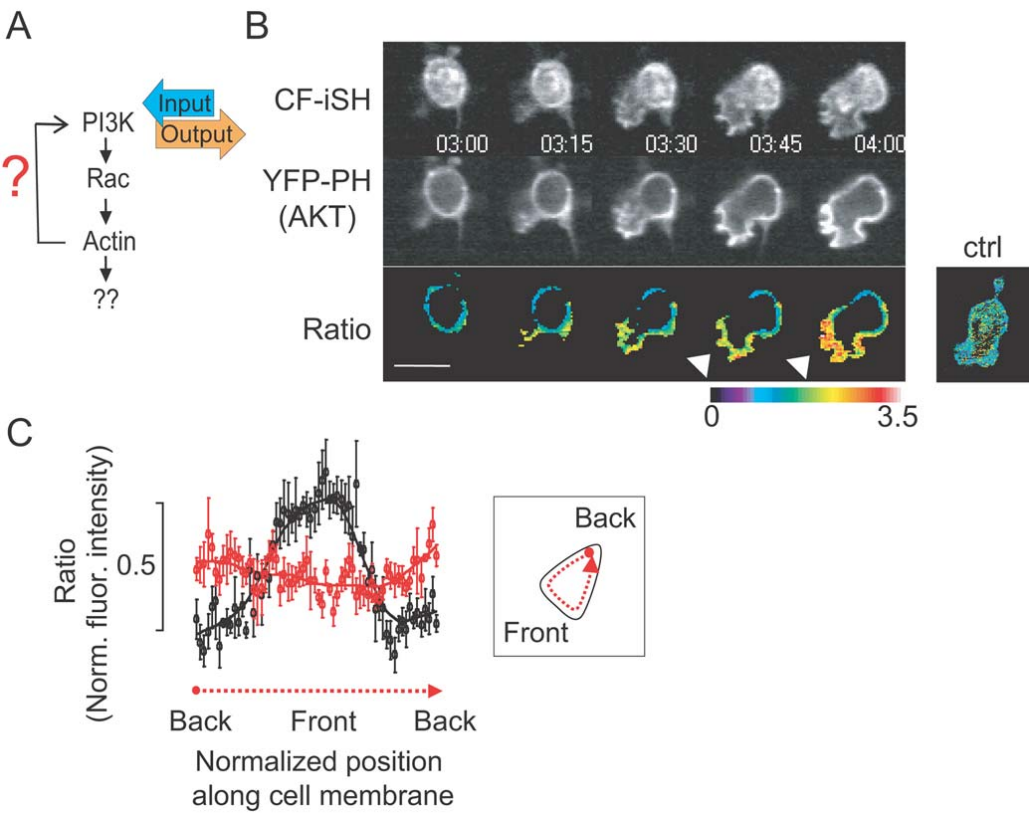
Delayed triggering of PIP_3_ polarization after an initial non-uniform increase in PIP_3_. (A) Schematic representation of experiments conducted in (B) and (C). (B) Time series analysis of PIP_3_ accumulation at the front of a migrating cell following uniform activation of PI3K in an unpolarized cell. Shown cell expresses the PI3K activator (CF-iSH, top row) and the PIP_3_ biosensor (YFP-PH(AKT), middle row). Bottom images were produced by ratioing the YFP-PH(AKT) images over the CF-iSH image. Note the higher ratio value at the pseudopod of the emerging polarized cells (arrowheads). The control panel on the right shows a ratio image of a cell expressing CF-iSH over a generic plasma membrane marker (myristoylated- and palmitoylated-YFP), arguing that CF-iSH is itself suitable as a uniform PM marker. Time unit; minute and second. (C) Line-scan analysis of the ratio images shown in (B) and of a control cell where a PM-YFP was again used instead of the YFP-PH(AKT). Normalized ratio values within a peri-plasma membrane region were calculated for the PI3K activated cells (analysis scheme is shown in inset). Black lines represent the normalized YFP-PH(AKT) distribution and red lines the control experiments showing that the normalization technique takes into account differences in membrane concentration. Error bars are given in S.E.M. (n = 5). Scale bars, 10 µm.

The observation that the activation of Rac failed to produce a phenocopy of the PI3K activation led us to rethink the logic of the positive feedback loop that drives the polarization and migration response. In order to understand the different roles of PI3K and Rac in polarization, we monitored the spatial distribution of PIP_3_ following uniform activation of either PI3K or Rac (Fig. 5A). By normalizing the concentration of PIP_3_-binding YFP-PH(AKT) over the CFP-conjugated membrane-anchored CF-iSH, we found that uniform activation of endogenous PI3K triggered first a uniform PIP_3_ distribution and then, with an approximately one minute delay, an enhanced PIP_3_ concentration at the leading edge of the polarized and migrating cell (Fig. 5B). The normalization of the CF-iSH over the generic plasma membrane marker, Lyn-YFP, confirmed that the observed asymmetric PIP_3_ accumulation was not due to membrane accumulation of CF-iSH at the leading edge (Fig. 5B, right small panel). A quantitative analysis of the normalized PIP_3_ gradients is shown in Fig. 5C. Together, these results support a model that PI3K triggers a self-enhancing reaction that amplifies PIP_3_ levels at the leading edge in the absence of upstream receptor signaling.

**Figure 6:**
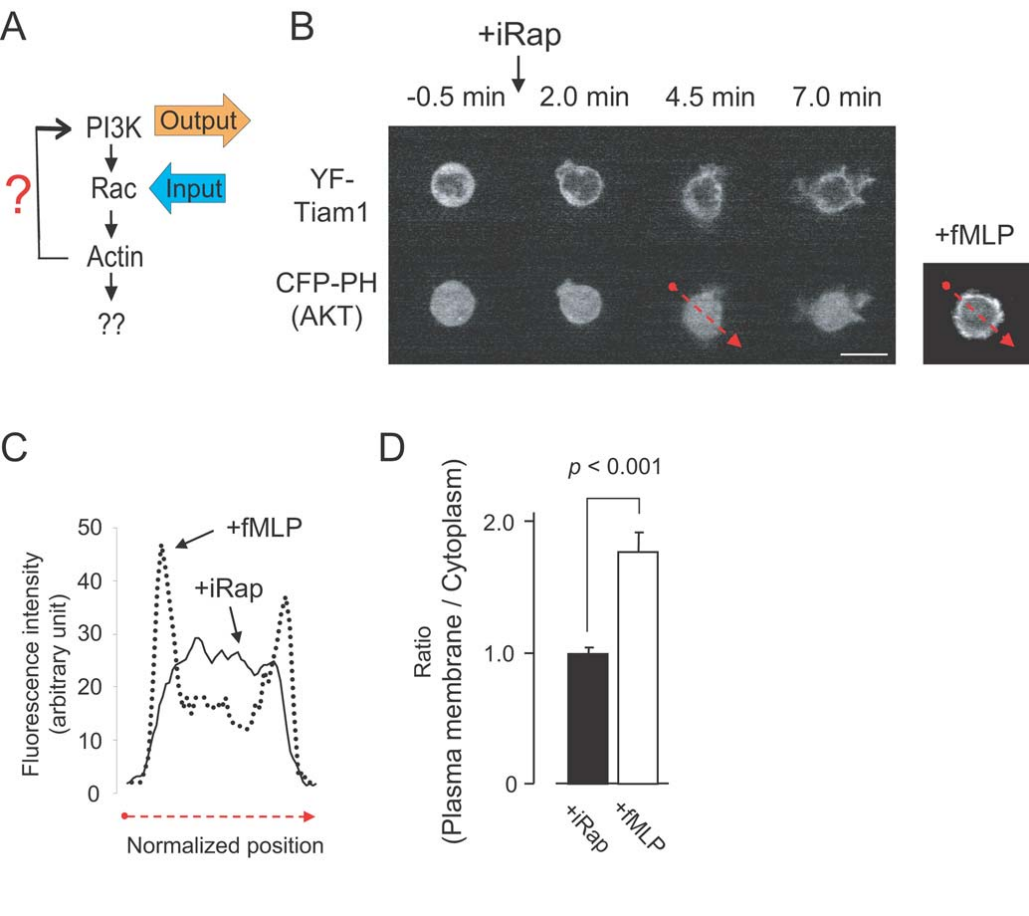
Endogenous Rac activation does not enhance PIP_3_ levels in cells. (A) Schematic representation of experiments conducted in (B) to (D). (B) Time series analysis showing that synthetic activation of endogenous Rac triggers pseudopod extensions but no measurable increase in PIP_3_ concentration. Top row shows the YFP-conjugated Rac activator construct (YF-Tiam1), and the bottom row the PIP_3_ biosensor CFP-PH(AKT). The panel on the right is shown as a control, demonstrating that cells induce a strong CFP-PH(AKT) translocation in response to fMLP. (C) Distribution of the YFP-PH(AKT) fluorescence intensity in (B) for a cross section through a cell where Rac has been synthetically activated (red dotted arrow in top panel). A comparison to a control cell is shown stimulated with fMLP instead of iRap. (D) Statistical analysis showing the ratio of the fluorescent intensity at the plasma membrane over the cytoplasm (lower right panel). Error bars represent S.E.M. (n>15 from more than three independent experiments). Scale bars, 10 µm.

We then tested whether Rac activation leads to dynamic PIP_3_ production as has been concluded from experiments where constitutively active Rac was expressed in neutrophils and other cell types [20]. In contrast to these earlier results, activation of endogenous Rac did not trigger PIP_3_ production measured by a translocation of CFP-PH(AKT) (Fig. 6A and 6B), even though the synthetic Rac activation was sufficient for actin-mediated pseudopod formation and ruffling. The bottom left panel in Fig. 6C shows a line scan analysis comparing the lack of a redistribution of CFP-PH(AKT) following Rac activation to the significant redistribution observed after fMLP stimulation. A statistical analysis of the lack of PIP_3_ production following Rac activation is shown in Fig. 6D. We confirmed the earlier report that over-expression of constitutively active Rac enhances PIP_3_ levels [20](data not shown), a result that we now believe may reflect the expected much higher concentration of active Rac enzyme due to the over-expressing of a constitutively active mutant protein. We conclude that activation of endogenous Rac at levels sufficient to trigger pseudopod extension are not sufficient to cause PIP_3_ production during the time window where polarization and migration is triggered.

**Figure 7:**
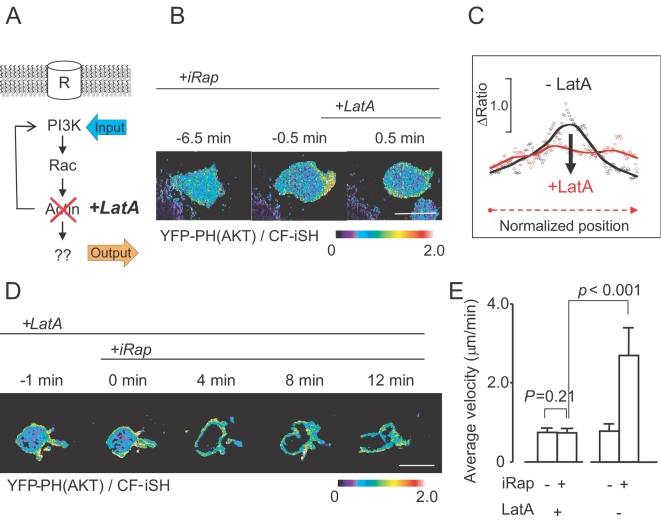
Actin polymerization is not needed for uniform but for polarized PIP_3_ production. (A) Schematic representation of experiments conducted in (B)–(E). (B) Ratio images (YFP-PH(AKT)/CF-iSH ) showing the suppression of polarized PIP_3_ accumulation in the front by LatA addition. LatA was added to cells in which PI3K was already synthetically activated. (C) Linescan intensity analysis along the plasma membrane region before (black line) and after (red line) LatA addition. (D) Time series showing that synthetic PI3K activation fails to generate polarized PIP_3_ in the presence of LatA. (E) Quantification of the imaging results, showing that LatA completely suppresses PI3K-induced cell migration. Error bars are given in S.E.M. (n = 15 from three independent experiments).

**Figure 8:**
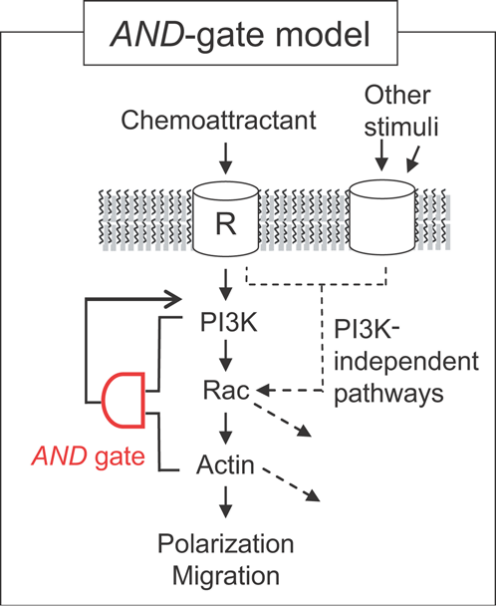
AND-gate model for PI3K-triggered cell polarization and migration. Coincidence detection model in which an AND-gate switch has to be triggered for cell polarization and migration. The requirement of the AND-gate for triggering PI3K-mediated polarization, may allow Rac, a common effector shared in different signaling pathways, to perform multiple functions including a role in PI3K-dependent and possibly in PI3K-independent cell polarization. Scale bars, 10 µm.

The inability of endogenous Rac activation to cause polarization also raised the question whether actin polymerization downstream of Rac is involved in the PIP_3_ polarization response (Fig. 7A). When we treated cells with Latrunculin A (LatA, an inhibitor of actin polymerization), polarized cells lost their enhanced PIP_3_ at the leading edge and stopped migrating (Fig. 7B). A quantitative line scan analysis more clearly shows this loss of polarization along the periphery (Fig. 7C). When LatA was added first, synthetic PI3K activation still triggered PIP_3_ production, but without triggering significant PIP_3_ polarization (Fig. 7D). Furthermore, PI3K-induced cell migration was completely suppressed (Fig. 7E). Cells occasionally showed irregularly-shaped, transient pseudopod extensions during Latrunculin A treatment that did not develop into a mature, polarized leading edge (Fig. 7D). A similar inhibition of migration and PIP_3_ polarization was observed in the presence of Cytochalasin D, a different type of inhibitor of actin polymerization (data not shown). This shows that PIP_3_ polarization and PI3K-mediated migration both require actin polymerization. However, Rac activation on its own, while triggering actin polymerization, induces neither polarization nor initiates the positive feedback to activate PI3K. This argues for a coincidence requirement for the local positive feedback loop so that a pathway from PI3K to Rac and polymerized actin as well as a second feedback loop from PI3K to PI3K that does not involve Rac are both needed for triggering PIP_3_ accumulation at the front (AND-gate logics; Fig. 8).

### Conclusions

We show that activation of endogenous PI3K is sufficient to trigger cell polarization and migration in the absence of upstream G-protein signaling. Given that chemoattractants activate PI3K, this provides a strong argument that endogenously present PI3K enzymes are sufficient as a physiological input for triggering cell polarization and migration. Our study further shows that the PI3K positive feedback module that can trigger cell polarization has the logical form of an AND gate that requires two pathways emanating from PI3K (Fig. 8). The existence of such an AND gate logic is based on the findings that the positive feedback for polarized PIP_3_ production is only triggered if PI3K is active and if Rac-mediated actin polymerization has occurred. Given that alternative pathways have been shown to exist for polarization and migration [28], [29], this AND gate has the interesting property that Rac-mediated actin polymerization can be employed by other cellular processes that rely on guanine exchange factors (GEF proteins) regulated by Ca^2+^ [30], [31], Wnt [32], integrins [33], [34] or other Rho GTPase members [35], [36] instead of PIP_3_. Together, our study provides a molecular explanation of how PI3K-dependent and independent polarization and migration mechanisms [28], [29] can exist in the same cell and how Rac can be used independent of cell polarization for alternative cell functions such as regulating cell adhesion and reactive oxygen signaling [37].

## Materials and Methods

### Materials

Synthetic biological probes, YF-iSH and YF-Tiam1, have been previously described [8], [9]. Briefly, YF-iSH consists of YFP, FKBP and an inter SH2 domain (420–615) of p85β (gene accession number: BC006796). YF-Tiam1 has a GEF domain (1012–1592) of Tiam1 (U16296) inserted into a YFP-FKBP backbone vector. These probes were always coexpressed with Lyn-FRB, a membrane targeted FRB, in order to induce iRap-mediated plasma membrane localization. The compound iRap was synthesized and provided by Tom Wandless's lab at Stanford [8], [9]. All the other chemical reagents were purchased from the following commercial companies: DMSO, Fibronectin, and fMLP from Sigma-Aldrich, Latrunculin A and Cytochalasin D from Calbiochem, DyeCycle from Invitrogen, and PTX from List Biological Laboratories.

### Methods

HL-60 cells (ATCC) were treated with 1.3% DMSO (Hybrimax, Sigma-Aldrich) for 3–5 days to induce neutrophil-like phenotypes [25]. Differentiated HL-60 cells were then harvested and electroporated in the presence of appropriate sets of DNA plasmids following the manufacturer's protocol (Nucleofection, Amaxa Inc.). After 4–8 hours in the cell culture conditions, the cells were plated on glass coverslips coated with fibronectin (50 µg/ml) for 30 minutes at room temperature. A Nipkow confocal fluorescent microscope was used to perform real-time, dual-color fluorescent imaging of adhered HL-60 cells. The description of the microscope equipment has been reported [8] with one additional piece: a UPlanSApo 60x objective lens (Olympus) in order to minimize chromatic aberration between CFP and YFP images. Alternate CFP-YFP images were taken every 15 seconds at room temperature with minimal laser intensity. Chemical reagents including iRap were applied manually to the medium with minimal shear stress to the cells. In experiments with PTX we treated differentiated HL-60 cells with the drug at 1 µg/ml for 6–8 hours prior to fluorescence imaging. All the imaging analysis was performed with Metamorph software (Molecular Devices). For statistical analyses, paired or unpaired student t-tests were applied depending on the type of experiment.

## Supporting Information

Movie S1 Synthetic PI3K activation induces cell polarization and migration. Note that the YF-iSH transfected cells (labeled with both green and blue) polarize and migrate whereas the untransfected cells (blue alone) do not. Blue; Vybrant DyeCycle (nuclei), Green; YF-iSH, Scale bar; 10 microm, Time unit; min:sec.
(6.36 MB AVI)

Movie S2 Synthetic Rac activation induces membrane ruffling but not cell polarization or migration. Note that the YF-Tiam1 transfected cells (shown in upper panel) exhibit membrane ruffling all over the cells whereas these cells did not show matured polarity or cell migration. Bottom panel: Vybrant DyeCycle (nuclei).
(3.21 MB AVI)
